# Supplementary material for: Identification of active sites on supported metal catalysts with carbon nanotube hydrogen highways
Source: Nat Commun. 2018 Sep 20;9:3827. doi: 10.1038/s41467-018-06100-9 (PMC6148247; doi:10.1038/s41467-018-06100-9)
Supplement: Supplementary file 1 — Supplementary Information [file 41467_2018_6100_MOESM1_ESM.pdf]

Supplementary Information for

Identification of Active Sites on Supported Metal Catalysts with Carbon Nanotube Hydrogen Highways

Nicholas M. Briggs[1], Lawrence Barrett[1], Evan C. Wegener[2], Leidy V. Herrera[1], Laura A. Gomez Gomez[1], Jeffrey T. Miller[2] and Steven P. Crossley[1]*

Affiliations:

[1] School of Chemical, Biological and Materials Engineering, University of Oklahoma, Norman, OK 73019

[2] Davidson School of Chemical Engineering, Purdue University, 480 Stadium Mall Drive, West Lafayette, IN 47907

correspondence to: stevencrossley@ou.edu

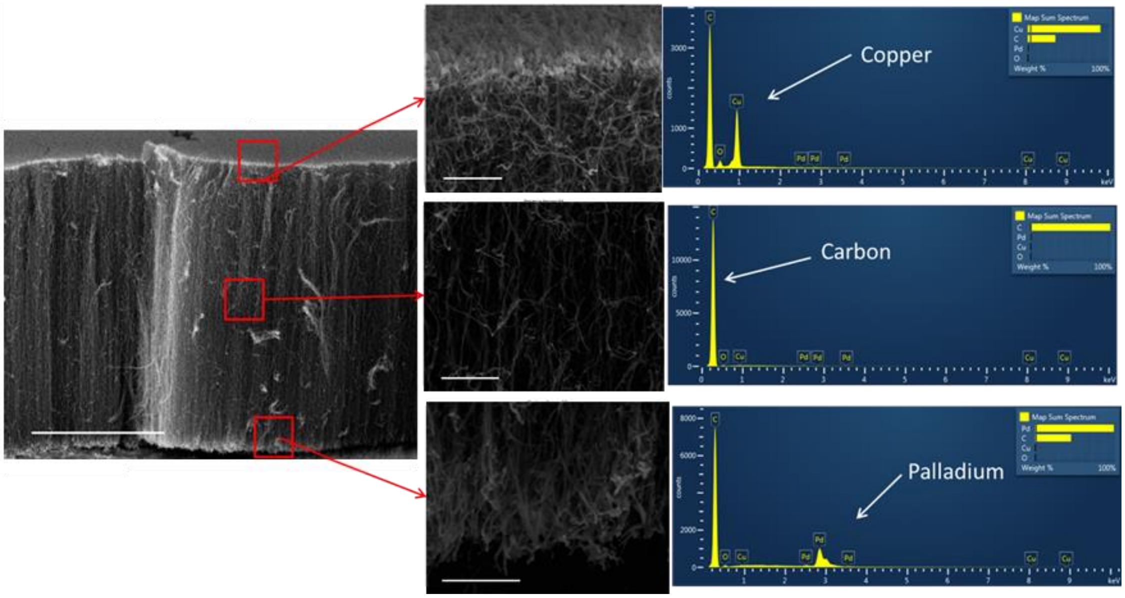

Supplementary Figure 1. SEM and EDS spectra of a nanotube forest with Pd and CuO deposited on each end through metal evaporation and after treatment in hydrogen for one hour at 400°C. Left image scale bar 25 microns, all three right scale bars are 1 micron.

Supplementary Discussion

<u>Pd and CuO separated on Carbon Nanotubes</u>

Pd and CuO on opposite ends of carbon nanotubes proven using SEM and EDS can be seen in Supplementary Figure 1. According to EDS data at the top of the nanotubes there is 72.2 wt% Cu and 0 wt% Pd, in the middle of the nanotubes there is 0 wt% Cu and 0 wt% Pd, and at the bottom of the nanotubes there is 0 wt% Cu and 68 wt% Pd. Thus indicating there are no Pd and Cu particles in contact.

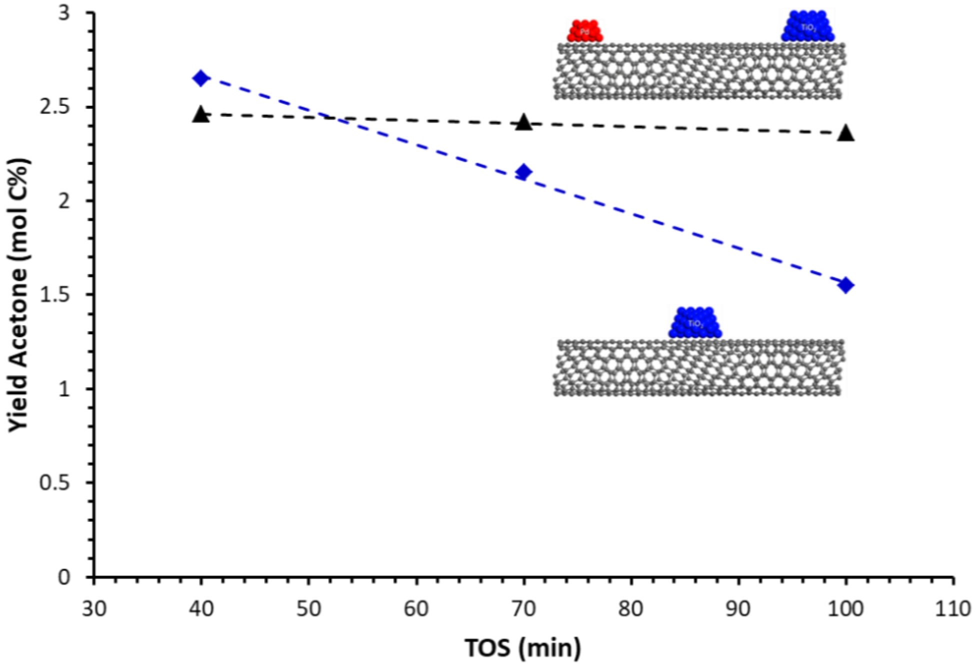

Supplementary Figure 2. Acetone yield comparison as a function of TOS between TiO_2/CNT, not calcined and Pd/CNT/TiO_2.

Supplementary Discussion

<u>Titania that has not been calcined after thermal evaporation.</u>

To further show the Pd is spilling over hydrogen which reaches the titania we made TiO_2/CNT, but did not calcine the sample in air. By not calcining in air more Ti is present and capable of reacting with acetone. As can be seen in Supplementary Figure 2 there are roughly the same number of active sites between the TiO_2/CNT and Pd/CNT/TiO_2 catalysts, but then as the reaction proceeded the yield of acetone drops, which is indicative of the consumption of the defects on the TiO_2/CNT catalyst. In contrast the Pd/CNT/TiO_2 catalyst maintains the yield of acetone and this is due to the Pd spilling over hydrogen and using the carbon nanotubes as a bridge to reach the TiO_2 to create more defects.

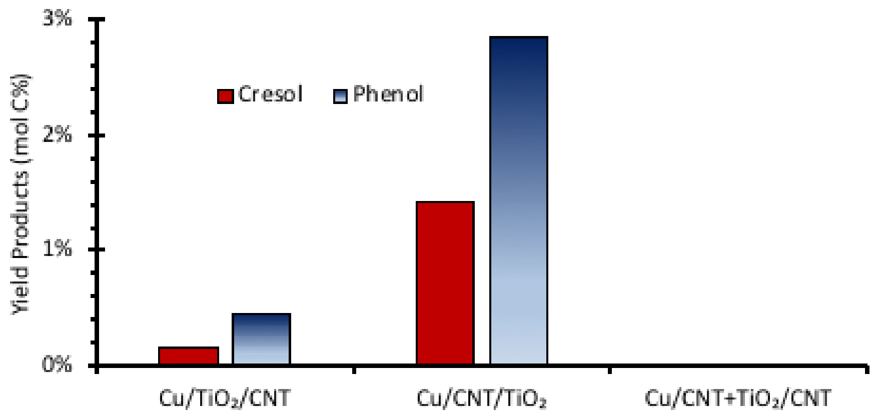

Supplementary Figure 3. Sum of quantifiable products (cresol + phenol) resulting from anisole conversion over Cu and TiO_2 catalysts supported on CNTs. T= 400 ^oC, P = 1 atm, under a H_2 flow, 100 min TOS.

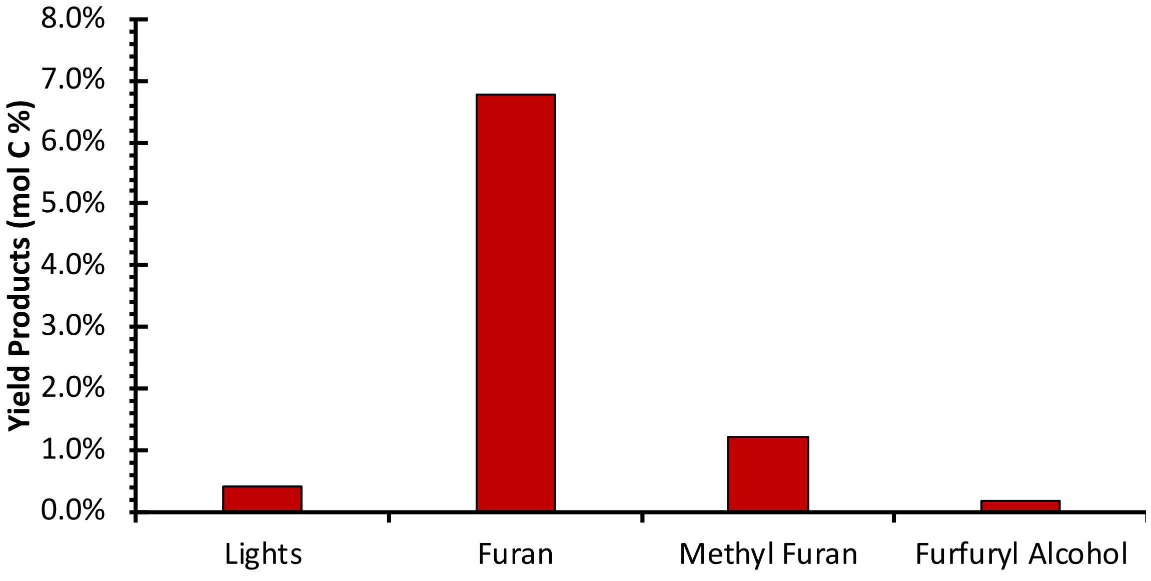

Supplementary Figure 4. Reaction feeding furfural over physical mixture Pd/CNT and TiO_2/CNT catalyst T= 400 ^oC, P = 1 atm, under a H_2 flow, 30 min TOS.

Supplementary Note 1. For physical mixture experiments (Pd/CNT + TiO_2/CNT), Pd and TiO_2 amounts in the reactor are equivalent to those present in the Pd/CNT/TiO_2 case presented in the manuscript in Figure .

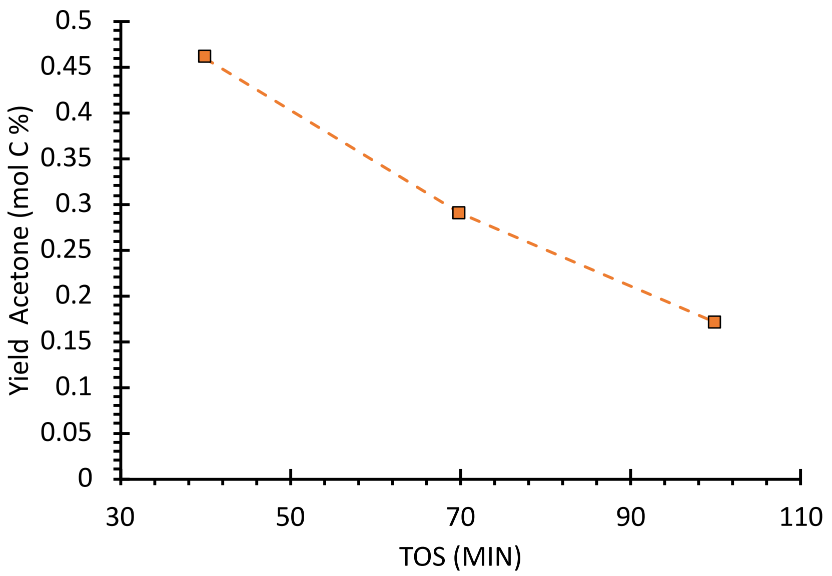

Supplementary Figure 5. Acetone yield when co-feeding furfural and acetic acid over physical mixture Pd/CNT and TiO_2/CNT At T= 400 ^oC, P = 1 atm, under a H_2 flow

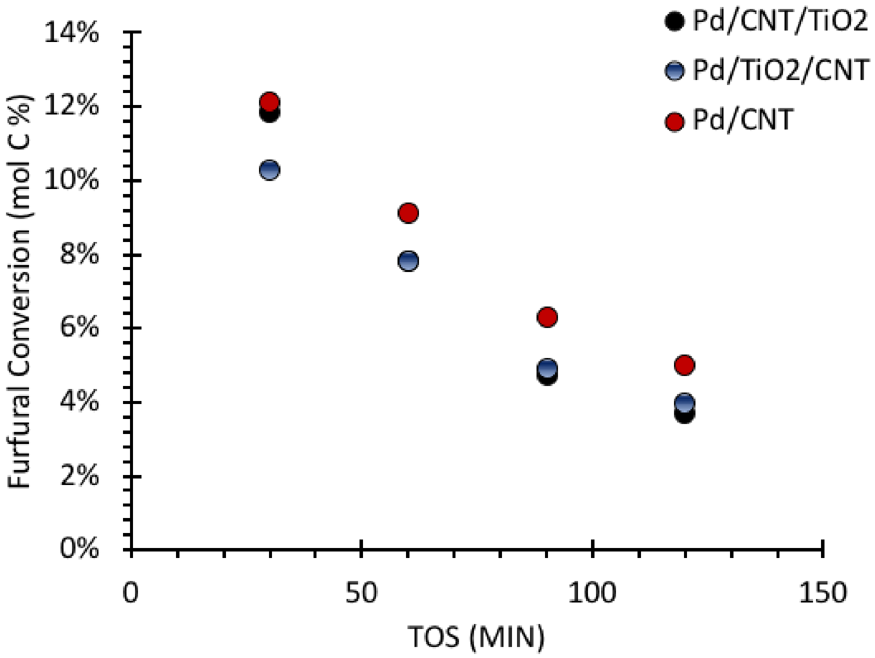

Supplementary Figure 6: Furfural conversion (estimated based on disappearance of furfural) over Pd and TiO_2 catalysts supported on CNTs. T= 400 °C, P = 1 atm, under atmospheric H_2 flow.

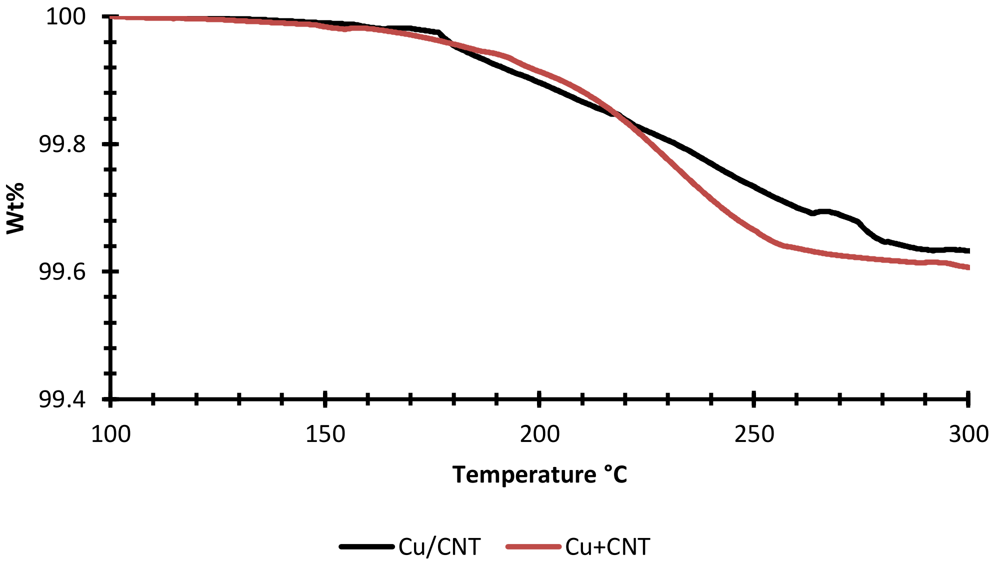

Supplementary Figure 7: TGA in a stream of 66vol% hydrogen in argon at a ramp rate of 3°C/min.

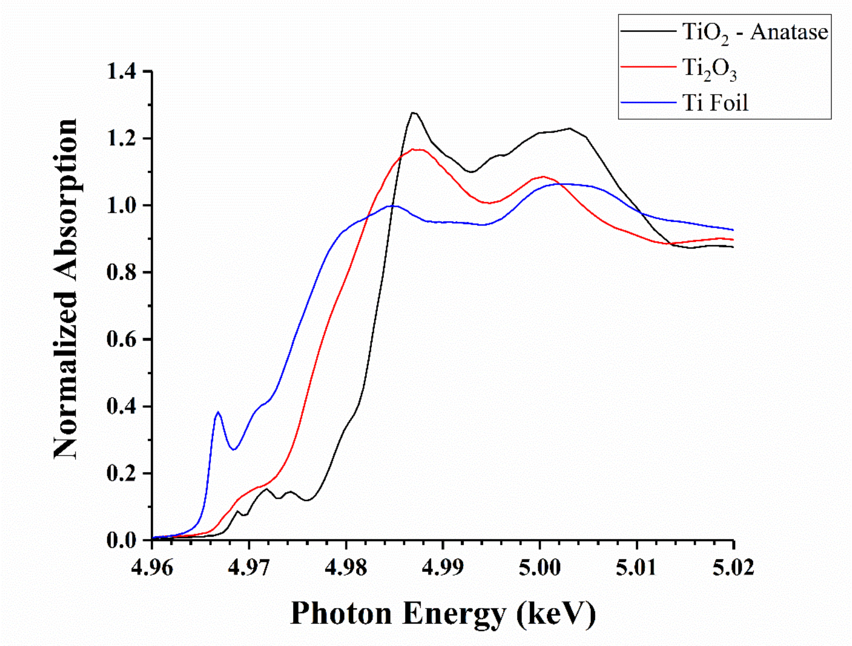

Supplementary Figure 8: Ti K edge XANES of Anatase (black), Ti_2O_3 (red), and Ti foil (blue). The edge energy, defined as the inflection point of the leading edge, increasing with increasing oxidation state.

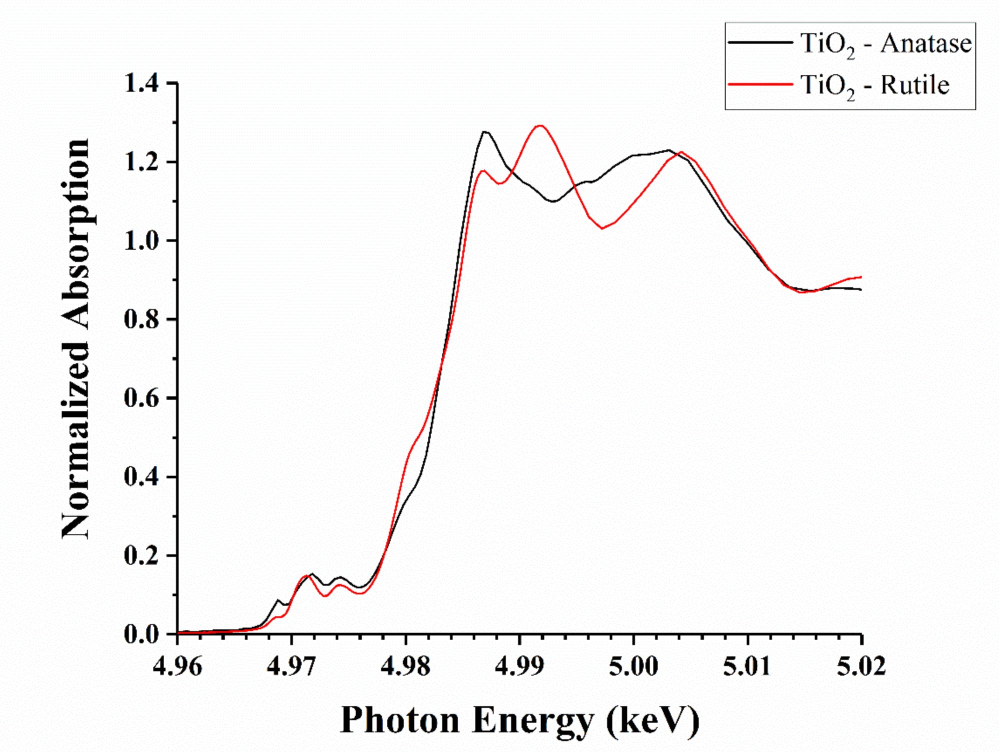

Supplementary Figure 9: Ti K edge XANES of the anatase (black) and rutile (red) phases of TiO_2. Although both phases are Ti(IV), differences in the coordination geometry of Ti in each phase result in differences in the XANES. The white line (initial feature after the absorption edge) of anatase consists of two peaks while rutile has three.

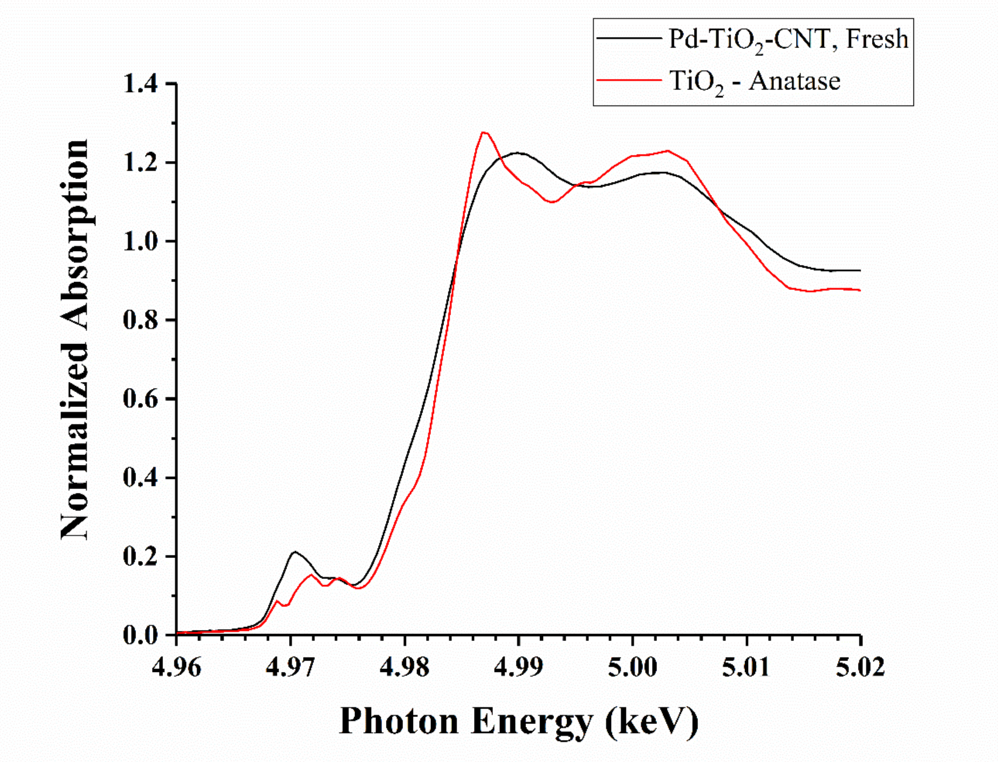

Supplementary Figure 10: Ti K edge XANES of fresh Pd-TiO_2-CNT (black) and anatase (red). The pre-edge peaks are consistent with the Ti being in the 4+ oxidation state and the two peaks observed in the white line of Pd-TiO_2-CNT indicates the sample is anatase. However, the peaks are broad and the ratio of the pre-edge peaks is different form the bulk sample. These features are consistent with the presence of very small TiO_2 clusters in the sample. The TiO_2-CNT and Pd-CNT-TiO_2 samples are very similar to Pd-TiO_2-CNT and similar conclusions can be drawn.

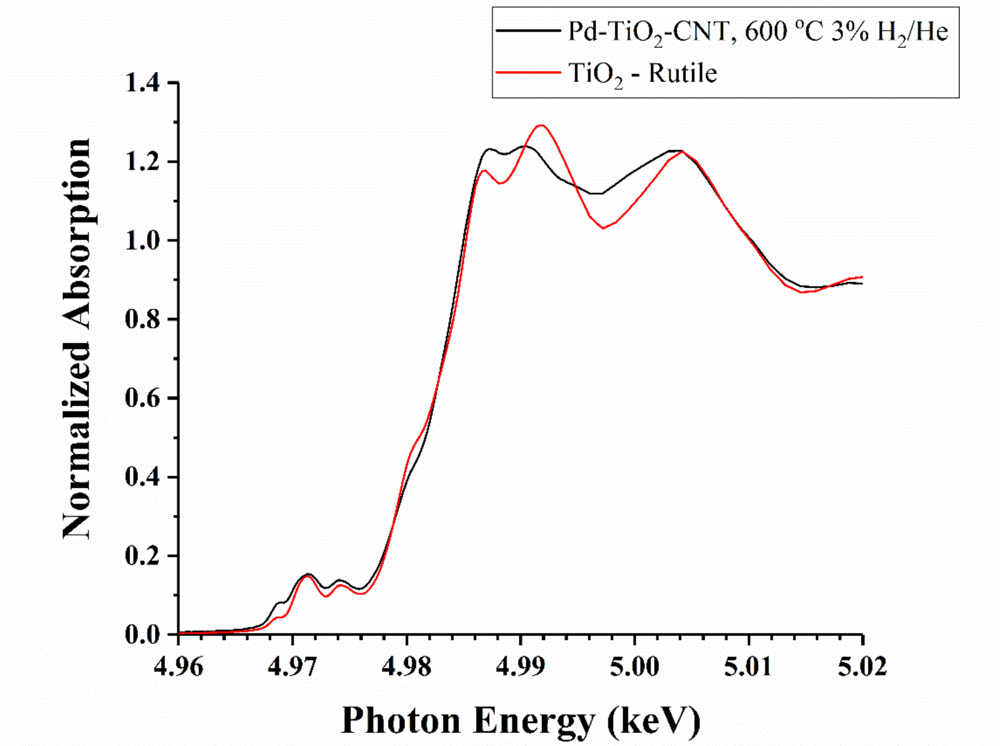

Supplementary Figure 11: Ti K edge XANES of Pd-TiO_2-CNT after reduction in 3% H_2/He at 600 ^{o}C (black) and rutile (red). The pre-edge peaks are consistent with the Ti being in the 4+ oxidation state. The sharper peaks in the white line compared to the spectrum of the fresh sample suggest that the TiO_2 clusters grow after heat treatment. The white line also shows three peaks (the first peak has begun to split into two) which is consistent with a fraction of the anatase undergoing a phase transformation to rutile. The TiO_2-CNT and Pd-CNT-TiO_2 samples are very similar to Pd-TiO_2-CNT and similar conclusions can be drawn.

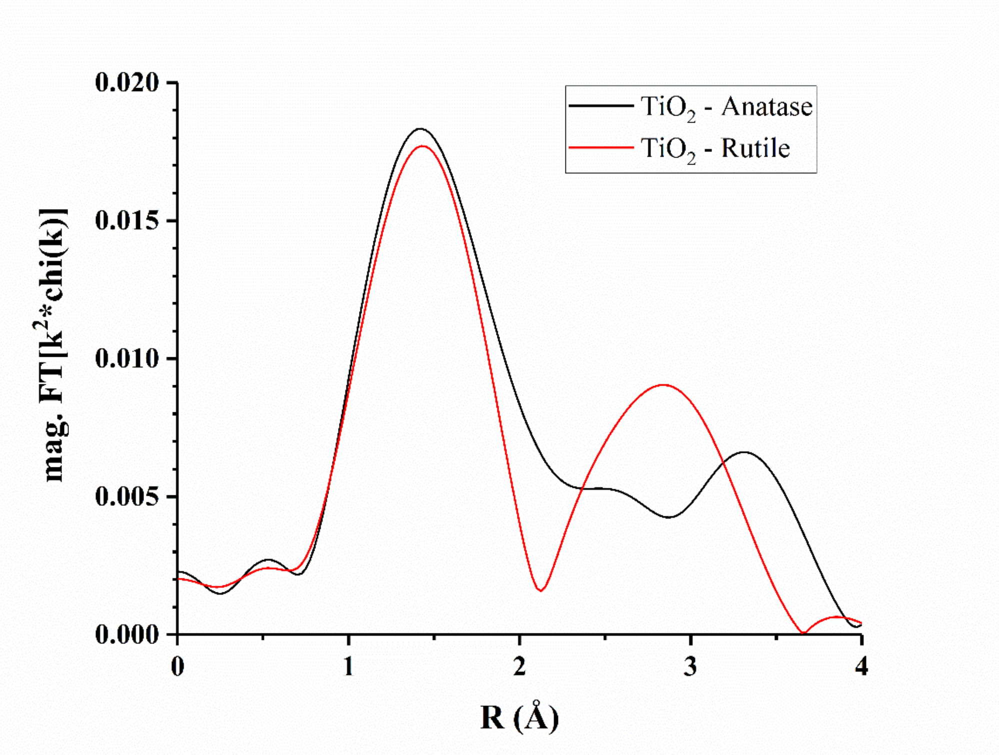

Supplementary Figure 12: The magnitude of the Fourier transform of the k^2-weighted EXAFS of anatase (black) and rutile (red). Both phases show a peak at 1.5 Å (phase uncorrected distance) from 6 Ti-O bonds and second-shell features between 2 and 4 Å (phase uncorrected distances) from Ti-O-Ti scattering. The differences between the two spectra results from the slight difference in the coordination geometry of Ti in each phase.

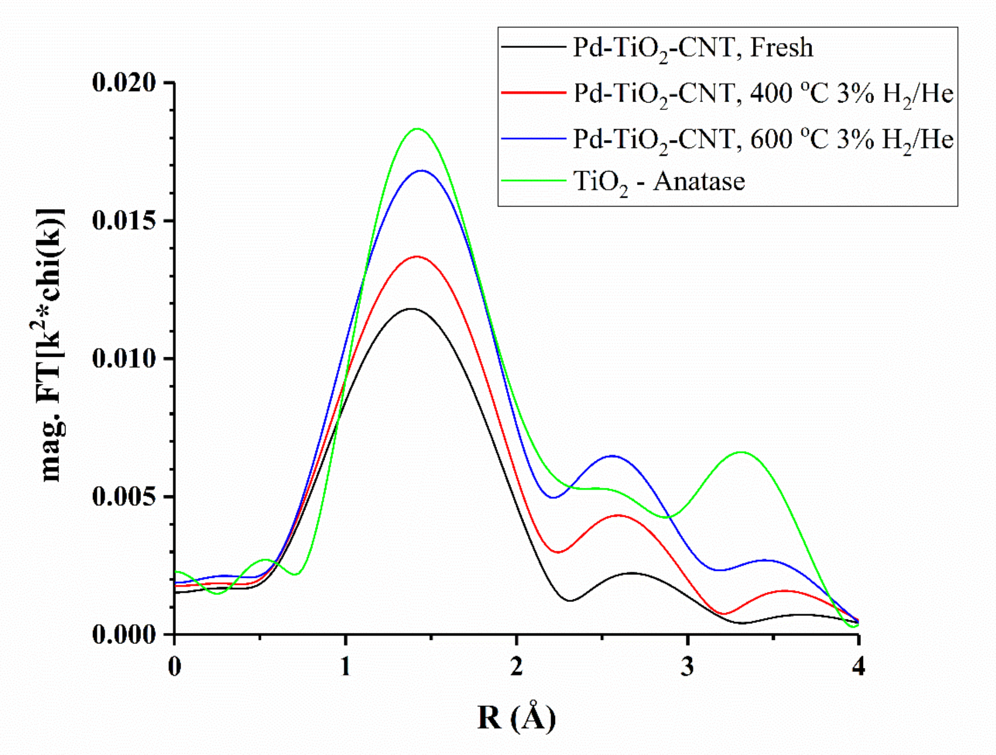

Supplementary Figure 13: The magnitude of the Fourier transform of the k^2-weighted EXAFS of Pd-TiO_2-CNT fresh (black), after reduction at 400 °C (red) and 600 °C (blue), and rutile (green). The decreased area of the peak at 1.5 Å (phase uncorrected distance) results from a Ti-O coordination number less than 6 and is consistent with the presence of small TiO_2 particles. This is also evidenced by the reduced second shell scattering peak in the sample compared to rutile. As the reduction temperature is increased the both the Ti-O and second-shell peak grow indicting a growth in the size of the TiO_2 clusters. The EXAFS results are consistent with XANES and the TiO_2-CNT and Pd-CNT-TiO_2 samples exhibit the same trends.

Supplementary Table 1.

Sample	wt% of Pd	wt% of Ti	wt% of Cu
Pd/CNT	0.29	0	N/A
TiO_2/CNT	0	0.60	N/A
Pd/CNT/TiO_2	0.24	0.66	N/A
Pd/TiO_2/CNT	0.29	0.76	N/A
CuO/CNT	0	N/A	1.08
Pd/CNT/CuO	0.26	N/A	1.01
CuO/TiO_2/CNT	0	0.66	1.10
Cu/CNT/TiO_2	0	0.66	1.08

Weight percent of metal deposited on CNTs as measured by EDS.

Supplementary Table 2.

Cu K Edge XAS Summary

Reference Samples							
Sample	Pre-edge Energy (keV)	Edge Energy (keV)	CN_{Cu-O}	R (Å)	$\Delta\sigma^2$ (*10^3)	E_o (eV)	Notes
Cu Foil	---	8.9790	12 (Cu-Cu)	2.556	0	0	Cu(0)
Cu_2O	---	8.9802	2	1.848	---	---	Cu(I)
CuO	8.9782	8.9834	4	1.957	---	---	Cu(II)
$Cu(Acac)_2$	8.9782	8.9841	4	1.92	0	0	Cu(II)

Supplementary Table 3.

Pd-CNT-CuO						
Sample	Edge Energy* (keV)	CN_{Cu-O}	R (Å)	$\Delta\sigma^2$ (*10^3)	E_o (eV)	Notes
Fresh	8.9808, 8.9854	3.5	1.94	1.0	-1.0	75% Cu(II) 25% Cu(I)
100 ^oC 3% H_2/He	8.9806, 8.9854	2.9	1.93	1.0	-0.9	45% Cu(II) 55% Cu(I)
150 ^oC 3% H_2/He	8.9806, 8.9854	2.7	1.92	1.0	-2.2	35% Cu(II) 65% Cu(I)
200 ^oC 3% H_2/He	8.9806, 8.9854	2.5	1.93	1.0	-1.1	25% Cu(II) 75% Cu(I)
300 ^oC 3% H_2/He	8.9790	6.0 (Cu-Cu)	2.51	2.0	-7.1	CN consistent with 1.5 nm NP’s

*First number is for Cu(I) feature and the second is for Cu(II)

Supplementary Note 2.

- XANES and EXAFS of the fresh sample show that the Cu in Pd-CNT-CuO is present as highly dispersed Cu oxide species, with the majority being present as Cu(II). However, a fraction is present as Cu(I). The fraction of each oxidation state is estimated from the Cu-O coordination number.
- As the reduction temperature is increased, Cu reduces from Cu(II) to Cu(I). After reduction at 300 ^{o}C metal particles are formed.

Supplementary Table 4.

Ti K Edge XAS Summary

Reference Samples							
Sample	Pre-edge Energy (keV)	Edge Energy (keV)	CN_{Ti-O}	R (Å)	$\Delta\sigma^2$ (*10^3)	E_o (eV)	Notes
Ti Foil	---	4.9660	---	---	---	---	Ti(0)
Ti_2O_3	---	4.9756	---	---	---	---	Ti(III)
TiO_2: Anatase	4.9688, 4.9718, 4.9743	4.9788	6	1.95	0.0	0	Ti(IV)
TiO_2: Rutile	4.9789, 4.9713, 4.9743	4.9793	---	---	---	---	Ti(IV)

TiO_2-CNT							
Sample	Pre-edge Energy (keV)	Edge Energy (keV)	CN_{Ti-O}	R (Å)	$\Delta\sigma^2$ (*10^3)	E_o (eV)	Notes
Fresh	4.9689, 4.9706, 4.9737	4.9790	4.8	1.93	3.0	-0.7	Ti(IV)
600 ^{o}C 3%H_2/He	4.9689, 4.9712, 4.9742	4.9792	5.7	1.94	1.0	-1.0	Ti(IV)

Pd-CNT-TiO_2							
Sample	Pre-edge Energy (keV)	Edge Energy (keV)	CN_{Ti-O}	R (Å)	$\Delta\sigma^2$ (*10^3)	E_o (eV)	Notes
Fresh	4.9689, 4.9706, 4.9737	4.9790	4.7	1.93	3.0	0.2	Ti(IV)
400 °C 3%H_2/He	4.9689, 4.9704, 4.9738	4.9791	5.2	1.93	3.0	-0.4	Ti(IV)
600 °C 3%H_2/He	4.9689, 4.9710, 4.9742	4.9792	5.3	1.94	1.0	-0.9	Ti(IV)

Pd-TiO_2-CNT							
Sample	Pre-edge Energy (keV)	Edge Energy (keV)	CN_{Ti-O}	R (Å)	$\Delta\sigma^2$ (*10^3)	E_o (eV)	Notes
Fresh	4.9689, 4.9704, 4.9737	4.9790	4.8	1.93	3.0	-0.4	Ti(IV)
400 °C 3%H_2/He	4.9689, 4.9707, 4.9741	4.9791	5.6	1.93	3.0	-1	Ti(IV)
600 °C 3%H_2/He	4.9689, 4.9713, 4.9742	4.9791	6.1	1.94	1.0	-0.7	Ti(IV)

Supplementary Note 3.

- The fresh samples are small anatase particles as evidenced by the two broad peaks observed in the white lines of XANES spectra. This is also evident from EXAFS which show Ti-O coordination numbers less than 6 (that of bulk TiO_2) and minimal second shell Ti-O-Ti scattering.
- As the samples are reduced at 400 and 600 °C the particles grow, and a fraction undergoes a phase transformation to rutile. This is seen in the XANES by the white line peaks becoming sharper and a third peak emerging. EXAFS also show a growth in size, i.e. Ti-O coordination numbers of 6 and increased second shell scattering.
- Only Ti(IV) is observed from the XANES, likely due to such a small fraction of the Ti reducing.
